# Whole-lake acoustic telemetry to evaluate survival of stocked juvenile fish

**DOI:** 10.1038/s41598-023-46330-6

**Published:** 2023-11-02

**Authors:** Alexander L. Koeberle, Webster Pearsall, Brad E. Hammers, Daniel Mulhall, James E. McKenna, Marc Chalupnicki, Suresh A. Sethi

**Affiliations:** 1 New York Cooperative Fish and Wildlife Research Unit, Department of Natural Resources and the Environment, Cornell University, Fernow Hall, Ithaca, NY 14853 USA; 2 New York State Department of Environmental Conservation, Division of Fish, Wildlife, and Marine Resources, Region 8 – Rochester/Western Finger Lakes, Avon, NY 14414 USA; 3 U.S. Geological Survey, Great Lakes Science Center, Tunison Laboratory of Aquatic Science, 3075 Gracie Road, Cortland, NY 13045 USA; 4 Aquatic Research and Environmental Assessment Center, Earth and Environmental Sciences, Brooklyn College, 123 Ingersoll Hall, Brooklyn, NY 11210 USA

**Keywords:** Conservation biology, Freshwater ecology, Population dynamics, Restoration ecology

## Abstract

Estimates of juvenile survival are critical for informing population dynamics and the ecology of fish, yet these demographic parameters are difficult to measure. Here, we demonstrate that advances in animal tracking technology provide opportunities to evaluate survival of juvenile tagged fish. We implemented a whole-lake telemetry array in conjunction with small acoustic tags (including tags < 1.0 g) to track the fate of stocked juvenile cisco (*Coregonus artedi*) as part of a native species restoration effort in the Finger Lakes region of New York, USA. We used time-to-event modeling to characterize the survival function of stocked fish, where we infer mortality as the cessation of tag detections. Survival estimates revealed distinct stages of juvenile cisco mortality including high immediate post-release mortality, followed by a period of elevated mortality during an acclimation period. By characterizing mortality over time, the whole-lake biotelemetry effort provided information useful for adapting stocking practices that may improve survival of stocked fish, and ultimately the success of the species reintroduction effort. The combination of acoustic technology and time-to-event modeling to inform fish survival may have wide applicability across waterbodies where receiver arrays can be deployed at scale and where basic assumptions about population closure can be satisfied.

## Introduction

Mortality is a key process governing population growth and features heavily in fisheries conservation and management, informing species ecology, harvest limits, and stocking programs^1,2^. Yet direct estimation of mortality is difficult to achieve, particularly with conventional marking approaches such as mark-recapture for small or juvenile fishes^3,4^. Juvenile fish survival is believed to be an important driver of population dynamics, where mortality through this critical period influences the trajectory of cohorts through later adult life stages^5,6^. Here, we demonstrate that contemporary biotelemetry technology and time-to-event modeling unlock new opportunities for survival modeling, even with small fishes. We applied whole-lake acoustic telemetry to track the fate of re-introduced juvenile cisco (*Coregonus artedi*) equipped with small transmitters (0.6 g and 3.5 g) in the Finger Lakes region of New York State, USA.

An important application of biotelemetry technology is to elucidate survival of juvenile stocked fish^7–9^. Releases of hatchery-reared fish are widely utilized to augment existing fish populations or to reestablish formerly extirpated or depleted populations^1^. Assessment of the fate of stocked fish, however, is typically lacking, and this information is needed to determine if augmentation efforts meet long-term conservation and management goals^3,10–12^. For example, mortality can provide information useful to manage fishery harvest rates and to assess whether stocking efforts can lead to a self-sustaining population^1,13^. Due to cost and logistical constraints, hatchery programs typically focus on juvenile life stage production, necessitating survival assessment approaches that can be applied to small fishes, such as small telemetry devices coupled with spatially extensive receiver arrays^7^. Stocking success, often evaluated as the contribution of hatchery fish to enhance wild fish stocks or the fitness of fish, is difficult to measure, and net positive effects of hatchery stocking have long been disputed^13^. Nonetheless, increased availability of approaches to estimate survival can in turn help hatchery managers evaluate and adapt stocking programs.

Biotelemetry uses electronic transmitters (hereafter referred to as ‘tags’) to track the movement and fate of study animals^14,15^. Technological advances in biotelemetry include tag miniaturization (e.g., tags < 1.0 g), longer tag and receiver battery life, and increased tag detection ranges^16,17^. Thus, while tag sizes previously restricted biotelemetry applications to relatively large specimens that can accommodate large telemetry tags with minimal adverse effects on survival, contemporary tags have been sufficiently miniaturized to provide ecological insight to a diverse age and size range of both freshwater and marine fish species^7,18,19^. Currently, the smallest biotelemetry tags are < 0.22 g injectable acoustic tags^20^.

Coupled with spatially extensive acoustic receiver arrays, acoustic tags provide the opportunity to estimate demographic parameters such as mortality^21^. If arrays are sufficiently dense to continuously detect tagged individuals, the fate of tagged fish can be determined. Assuming that tagged fish move throughout their environment and in systems closed to immigration and emigration, mortalities are reflected by two detection signals. First, the cessation of tag detections, within the period of active tag battery life, can indicate a fish perished in regions outside the detection range of receivers. Second, continuous tag detections at a single receiver for a repeat time interval can indicate a fish perished within the detection range of a receiver^9^. Acoustic tags and spatially extensive receiver arrays can therefore yield so called ‘time-to-event’ data whereby both the status of a tagged fish (alive or dead) and the time of mortality since release can be inferred^22,23^. Time-to-event data enable powerful mortality modeling approaches that can characterize the survival function of tagged fish^24^. The survival function provides information on the time path of mortality and can accommodate the influence of subject-level covariates^25^. Thus, in addition to estimating survival up to any point in time, time-to-event modeling can be used to understand the influence of biological and environmental covariates on survival.

In recent years, native coregonine (*Coregonus* spp.) restoration has been attempted by fishery scientists and managers to improve ecosystem integrity throughout the Laurentian Great Lakes basin of North America^26–28^. In the Lake Ontario basin, cisco are a pelagic cold-water species that was historically a major component of the native forage fish assemblage and had high commercial, cultural, and recreational significance to coastal communities^29^. Cisco populations have sharply declined throughout the basin due to a combination of fishing pressure, habitat and water quality degradation, and competition from non-native aquatic species^30–32^. Some ongoing cisco restoration efforts involve reintroductions or augmentations from hatchery stocking programs; however, post-release survival rates are not well-described for this species in the Great Lakes region^32,33^. This lack of survival information on stocked cisco currently hampers both the ability to assess stocking success and to adjust stocking practices to achieve conservation, restoration, or management targets for this species.

In this study, we track the fate of hatchery-reared juvenile cisco reintroduced into Keuka Lake, New York, USA. The fate of a subset of released fish equipped with acoustic tags were monitored through an extensive lake-wide array of acoustic receivers which autonomously logged detections as tagged fish swam within the detection range of a receiver. We infer that the cessation of tag detections on the array for a given tagged fish indicates a mortality event. Our objectives were to: (1) Implement a whole-lake scale passive acoustic telemetry array to assess the fate of acoustically-tagged fish, (2) Analyze the individual fates of tagged cisco using time-to-event models that characterize the time path of survival for released fish, and (3) Interpret stocked fish survival information to support assessment and adaptation of stocking efforts to restore extirpated cisco in Keuka Lake. Our results indicate mortality events could be clearly discerned for juvenile cisco using tag detections, meeting conditions for time-to-event survival modeling. This study suggests that contemporary acoustic biotelemetry may have wide applicability in estimating survival of small fish in waterbodies with sufficient receiver coverage and that meet basic assumptions about population closure.

## Methods

### Study system

**Figure 1:**
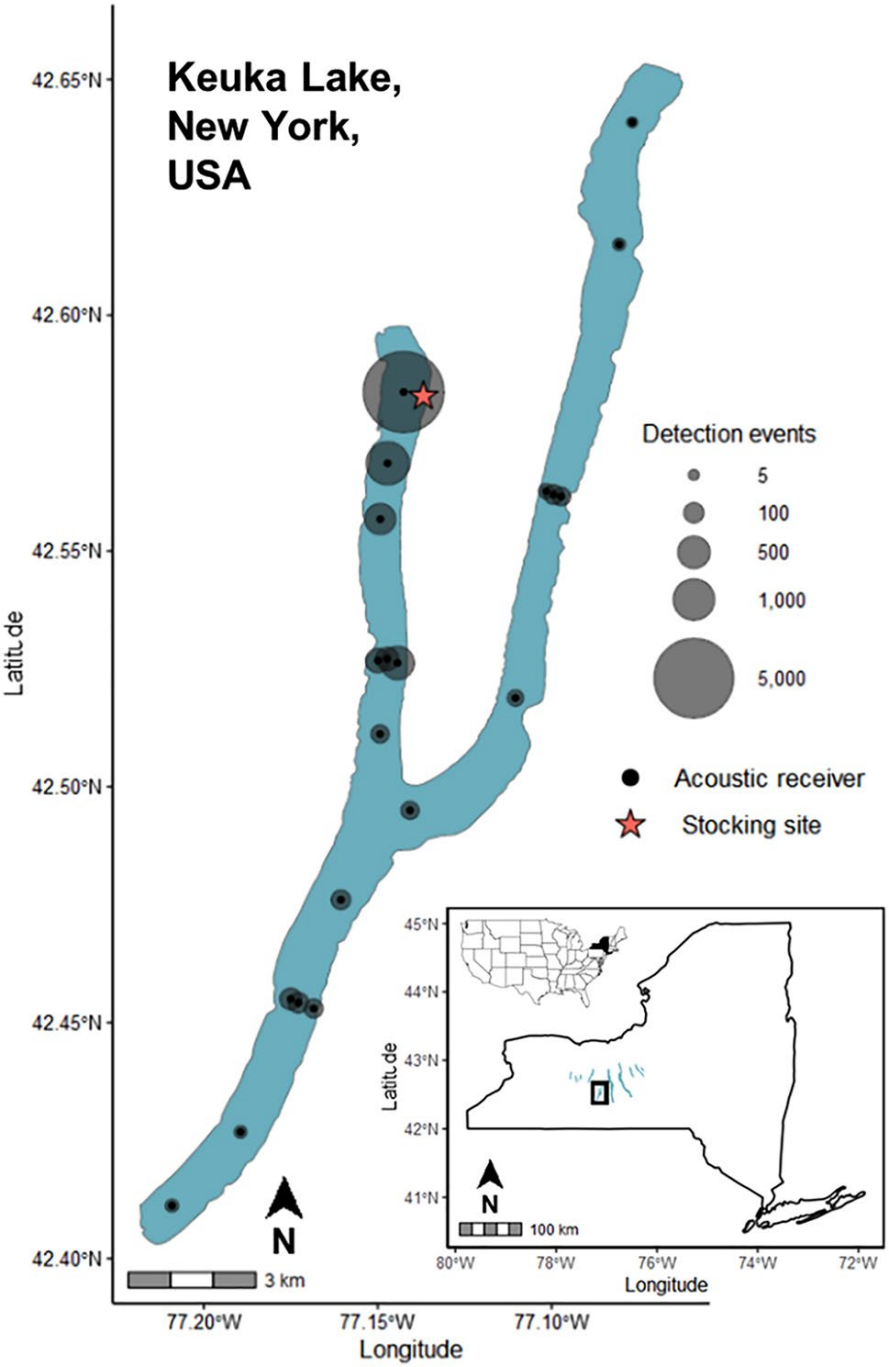
Study location. Study location of a whole-lake acoustic telemetry study to track survival rates of stocked juvenile fish in Keuka Lake, New York, USA. The solid black circles show acoustic receiver placement for whole-lake level array coverage and the transparent gray circles show the total number of ‘detection events’ (discrete events of individual tag detections) from October 2019 to August 2021. The inset map (bottom right) indicates the Finger Lakes region of New York State, USA.

Keuka Lake is an inland waterbody located in the Finger Lakes region of New York, USA and is within the Lake Ontario basin of the Laurentian Great Lakes. Known for its distinct ‘y-shape’, Keuka Lake has a 57 m maximum depth, 31 m mean depth, 3 km maximum width, and a total surface area of 46.88km^2^ (Fig. 1)^34^. Keuka Lake also contains a popular native lake trout (*Salvelinus namaycush*) fishery. In addition to providing an important fishery resource in Keuka Lake, the lake trout population is unique within the region as it is self-sustained through ‘wild’ recruitment, whereas most other lake trout populations in the region are supported with hatchery augmentation. Thus, maintaining lake conditions to conserve lake trout recruitment in Keuka Lake is a management priority by New York State Department of Environmental Conservation (NYSDEC). Cisco were historically the dominant prey fish species for predators like lake trout, however competition with introduced, non-native alewife (*Alosa pseudoharengus*) and rainbow smelt (*Osmerus mordax*) and degraded water quality from high nutrient input led to their extirpation in Keuka Lake by the 1990s. In recent years, non-native fish populations have declined, and water quality has improved from nutrient management as the lake is undergoing oligotrophication. Resource managers hypothesize that future limnological conditions favor cisco over non-native alewife, and therefore seek to bolster fish community resilience by reintroducing cisco to restore this native species^28^.

### Acoustic-tagging and hatchery releases

**Figure 2:**
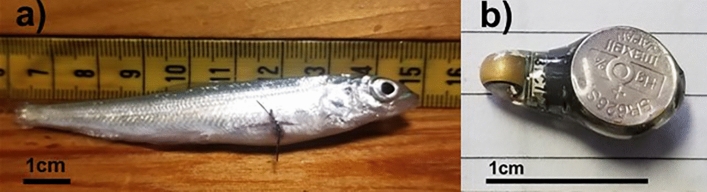
Acoustic-tagged juvenile fish. Juvenile cisco (*Coregonus artedi*) re-introduced from extirpation in Keuka Lake, New York, USA. (**a**) Juvenile cisco with a surgically implanted acoustic transmitter. (**b**) A small (0.6 g) acoustic telemetry transmitter used for age-0 fish in this study. Photographs by M. Chalupnicki, USGS.

From 2019 to 2020, 296,979 juvenile cisco were stocked from regional hatcheries into Keuka Lake across multiple release cohorts (Table 1). We tagged and released 210 juvenile cisco of two age classes (10 months post hatching, hereafter referred to as age-0; and 19 or 22 months post hatching, hereafter referred to as age-1) with Juvenile Salmon Acoustic Telemetry System (JSATS) Acoustic Micro Transmitters (AMT; Fig. 2; Table 1). These are small acoustic tags that emit uniquely coded sound pulses on pre-programmed frequency and time intervals to track individual fish across a fixed acoustic receiver array^7^. Age-0 fish were surgically implanted with 0.6 g tags, and age-1 fish were implanted with 3.5 g tags. All experimental protocols for sampling and handling of fish were approved by U.S. Geological Survey and the New York State fish collections review office. Fish collections occurred under Scientific License to Collect or Possess Permit #2977 and were in accordance with all fish handling, animal care, and ethical use requirements governing fish collection permits as granted by NYSDEC. Additionally, all methods for this study are reported in accordance with Animal Research: Reporting of In Vivo Experiments (ARRIVE) guidelines. Acoustic tag implantations followed protocols described by McKenna et al.^33^ that would lead to low to no tag-related mortality, rather than strictly following a 2% tag size to body size rule. This protocol included holding all tagged fish onsite for at least 14 days before release to ensure tag retention and to monitor their post-surgery survival and condition. We did not observe any mortalities or tag expulsions among study fish prior to their release into Keuka Lake, consistent with findings by McKenna et al.^33^. All acoustic transmitters were programmed with a 20-s transmission interval (e.g., ‘ping rate’). At this ping rate, we conservatively anticipated a minimum expected battery lifespan of 262 days for the 0.6 g tags and 1218 days for the 3.5 g tags (80% of full battery life according to manufacturer specifications). See SI 1.1 for further information on surgery procedures and tag specifications.

**Table 1:** Summary of hatchery-reared juvenile cisco (*Coregonus artedi*) stocked in Keuka Lake, New York, USA from October 2019 to October 2020.

Cohort (age)	Release date	Total fish stocked	n tags (tag size)	Age-at- release (months)	Mass (g)	Total length (mm)
Fall 2019 (1)	9-Oct-2019	28	28 (3.5 g)	Age-1 (22)	111.1 ± *24.0* (46.7–156.6)	221.9 ± *15.6* (183–252)
Fall 2019 (0)	9-Oct-2019	92,225	60 (0.6 g)	Age-0 (10)	9.5 ± *1.8* (5.5–13.9)	111.1 ± *6.6* (98–136)
Summer 2020 (1)	7-July-2020	260	56 (3.5 g)	Age-1 (19)	53.5 ± *10.6* (34.4–75.7)	184.4 ± *9.6* (165–204)
Fall 2020 (0)	15-Oct-2020	204,466	66 (0.6 g)	Age-0 (10)	12.7 ± *1.7* (9.9–16.0)	116.8 ± *5.4* (105–128)
Total		296,979	210			

Age-0 fish, also known as Fall Fingerlings, are 10-months old from hatching whereas age-1 fish, known as Yearlings, are between 1 and 2-years old. Tagged cisco mass (g) and total length (mm) are shown for each cohort as mean ± *SD* (range).

#### Acoustic receiver array

We configured 24 acoustic receivers throughout Keuka Lake to form a lake-wide telemetry array to maximize spatial coverage for detecting post-release movements and the fate of tagged cisco after stocking (Fig. 1). We deployed acoustic receivers (Lotek-brand Wireless Hydrophone System 4250 Series), rated to 400 m depths, with four lithium primary D-cell batteries to increase operation lifespan. Each receiver was serviced every three to four months to download autonomously logged data of tag detections and to refresh lithium batteries. Prior to releasing tagged fish, we quantified receiver efficiency by assessing the detection of study tags at increasing distances from moored receivers in the lake. We found that acoustic receivers had a linear diameter detection range of approximately 200 to 300 m (see SI 1.2 for further acoustic receiver specifications, range testing, and receiver array details).

Acoustic receiver moorings were anchored on the lake bottom for the duration of the study, with all receiver units facing upward in the water column (Fig. S1). Because cisco are a pelagic cold-water species, we expected tagged fish to primarily inhabit deep waters that range from below a thermocline, which forms between 9 and 11 m during summer months, to depths near the bottom of Keuka Lake (fish could occupy depths up to approximately 57 m)^31,35^. Hence, we did not suspect deflection or interference of tag transmissions from seasonal thermal strata due to their habitat preference^36^. Additionally, three acoustic receivers were stationed midway in each branch to increase coverage for offshore and nearshore movements of tagged cisco between lake branches. We deployed a receiver upstream (stream wetted width = 20 m) within the Keuka Lake outlet and a receiver downstream (stream wetted width 100 m) at the confluence of Seneca Lake to determine if any tagged fish emigrated from the study system (Fig. S1). All tagged and untagged cisco in this study were stocked offshore via boat in the northwestern branch (Fig. 1).

### Detection filtering

As pelagic schooling fish, cisco move widely^37^. In Keuka Lake, we expected that surviving cisco would be capable of moving among branches at a lake-wide scale and at deep depths where receivers were moored. Thus, we assumed that if a tagged fish was alive, it would be detected on the whole-lake acoustic receiver array; in contrast, we inferred a mortality event when a tagged fish ceased to be detected by any receiver across the array. All datafiles downloaded from acoustic receivers were first processed with acoustic data processing software (Lotek WHS Host software; V1.8.3942.3) to remove detections that did not correspond with a fish tag identification (hereafter tag ID), i.e., false detections^9,38^. Subsequent detection datafiles were post-processed using a combination of custom filtering scripts, detection functions (‘GLATOS’ package^39^ in program R; version 4.2.2, R Core Team 2022), and manual inspection of each tagged fish’s data to obtain detection histories.

To distinguish true positive detections from spurious detections on receivers, we removed detections that (1) did not correspond to a known tag ID, (2) occurred past the expected tag battery lifespan, (3) failed to meet the minimum requirements for consecutive detections (required ≥ two detections on the same receiver ≤ 4-min apart), and (4) occurred outside a 20-s transmission rate window (± 2-s at each 20-s multiple within 4-min). These criteria, including the 4-min interval, were determined from the tag transmission rate and receiver detection range, inferred swimming behavior of this species, as well as detection criteria from studies with similar telemetry equipment^39–42^. We retained 19.3% of total detections using these criteria for a final dataset of true tag detections. To visualize detections across the receiver array, we collapsed individual detections into discrete ‘detection events’ for each tagged fish to account for arrival and departure at a receiver location^39^. Detection histories for each fish revealed when tagged fish ceased to be detected, i.e., died outside the detection range of receivers. Once a tagged cisco went undetected, we inferred mortality as a function of its last known time detected and a detection interval. For all tagged cisco observed on the receiver array, we calculated the average time between observed detections for individual fish. Mortality, or inferred time-to-death, was therefore calculated as the last time detected on the array plus half the average time interval between tag detections. For further information on our detection filtering approach for this study see SI 1.3.

### Modeling assumptions

Lake-wide acoustic receiver deployment provides the opportunity to track tagged cisco across their lifespan as ‘time-to-event’ data, where both the outcome of interest (death or censorship) and the event time (days after release) are known^25,43^. For time-to-event models of biotelemetry data, we assumed that the status of an individual animal is always known and that survival probabilities are equal for censored and uncensored individuals^43,44^. To meet assumptions for subsequent survival analyses, we inferred that all tagged fish have an equal probability of detection across the receiver array (no systematic detection heterogeneity). Further, we assumed that the acoustic-transmitter burden does not affect survival of tagged fish, as supported by hatchery cisco survival and tag retention trials^33^ such that survival estimates from tagged fish represent population-level survival rates. Lastly, some tagged fish could be consumed by a piscivorous predator, and the tag could therefore be at large for some time period in the predator’s stomach before being excreted. Estimated predator tag retention time varies by tag size, and false detection rates have been reported^9^. We assumed that the two different sized tags, which were ≤ 3.5 g and used in this study, would likely be excreted within one to three days. This is consistent with existing literature of comparable tag sizes^38,45,46^ and would thus have a low impact on inferred survival times.

To meet assumptions for tracking the lifespan of tagged study fish, we assume Keuka Lake is a closed system. Because cisco were extirpated and stocked fish did not have sufficient time to reach reproductive maturity, we assumed no cisco other than those stocked entered the system. Closed population status was confirmed by a lack of tag detections at receivers positioned in the exit point (Keuka Outlet; Fig. S1). Combined, the lack of additions and non-mortality related losses imply that estimated mortality rates reflect true cisco survival, uncontaminated by immigration, emigration, or recruitment.

### Survival analysis

A major challenge in quantifying survival rates of stocked fish is detecting individuals over the period of their life history under study. For example, conventional mark-recapture approaches for estimating apparent survival such as Cormack-Jolly-Seber models^47,48^ necessitate repeated captures of individuals and require estimation of both detection processes and survival processes^8,21^. A spatially extensive whole-lake acoustic array coupled with continuously transmitting tags provides time-to-event data whereby status is inferred by tag detections (alive) or cessation of detections (dead). Accordingly, probability of detection across the receiver array is equal to one because the fate of tagged fish is known.

We used two time-to-event modeling approaches to evaluate survival of stocked juvenile cisco. First, we visualized empirical summaries of survival outcomes using Kaplan–Meier curves^49^. Subsequently, we applied Cox proportional hazards models to characterize the survival function of juvenile stocked cisco and evaluate whether subject-level covariates influence fish survival^25,50^. The primary output of Cox proportional hazards modeling is a survival function, *S(t)*, which provides predicted survival rates up to a specified time, *t*, and which can incorporate covariate effects on survival if available. Through survival functions, time-to-event model approaches such as Cox proportional hazards models provide the ability to estimate survival up to any desired time while also providing information on the shape of the survival rate over the study period. Right censorship is a common feature of time-to-event data, whereby the observation period on a given subject terminates prior to that individual experiencing mortality^21,25^. Time-to-event models can accommodate censorship events; however, no fish in this study survived to their expected tag battery lifespan or past the end of acoustic receiver monitoring. Thus, right censorship events did not contribute to our survival estimates.

We considered a suite of covariates that could affect juvenile stocked cisco survival rates. Stocked fish covariate data were collected before release and included total length (mm), mass (g), and age-at-release (age-0 or age-1). We used the mean cohort mass and total length for one fish with missing size measurements from Fall 2019 (age-0 fish). We also estimated Fulton’s body condition factor (*K*) as 100,000 × mass × length^-3^^51^. Although survival rates may vary by sex, juvenile cisco cannot be reliably sexed from visual observation, so this information was not available for modeling. We tested for collinearity among variables and found positive correlation between age, length, and mass. Consequently, we explored models with age or size (mass or length) metrics, but not both as an interaction. We did not detect correlation between age and *K*. Length and mass were measured at release and, along with *K*, were not treated as time-varying covariates for survival analyses. We explored the support for covariate effects using multi-model inference based on Akaike’s Information Criterion adjusted for small sample size (AICc)^52^. Thus, we fit 17 total Cox proportional hazards models to the time-to-event dataset of tagged fish released from October 2019 to October 2020, including release year, age-at-release, length, mass, and *K*, along with interaction terms that allowed for testing size effects within age cohorts.

## Results

### Whole-lake biotelemetry

**Figure 3:**
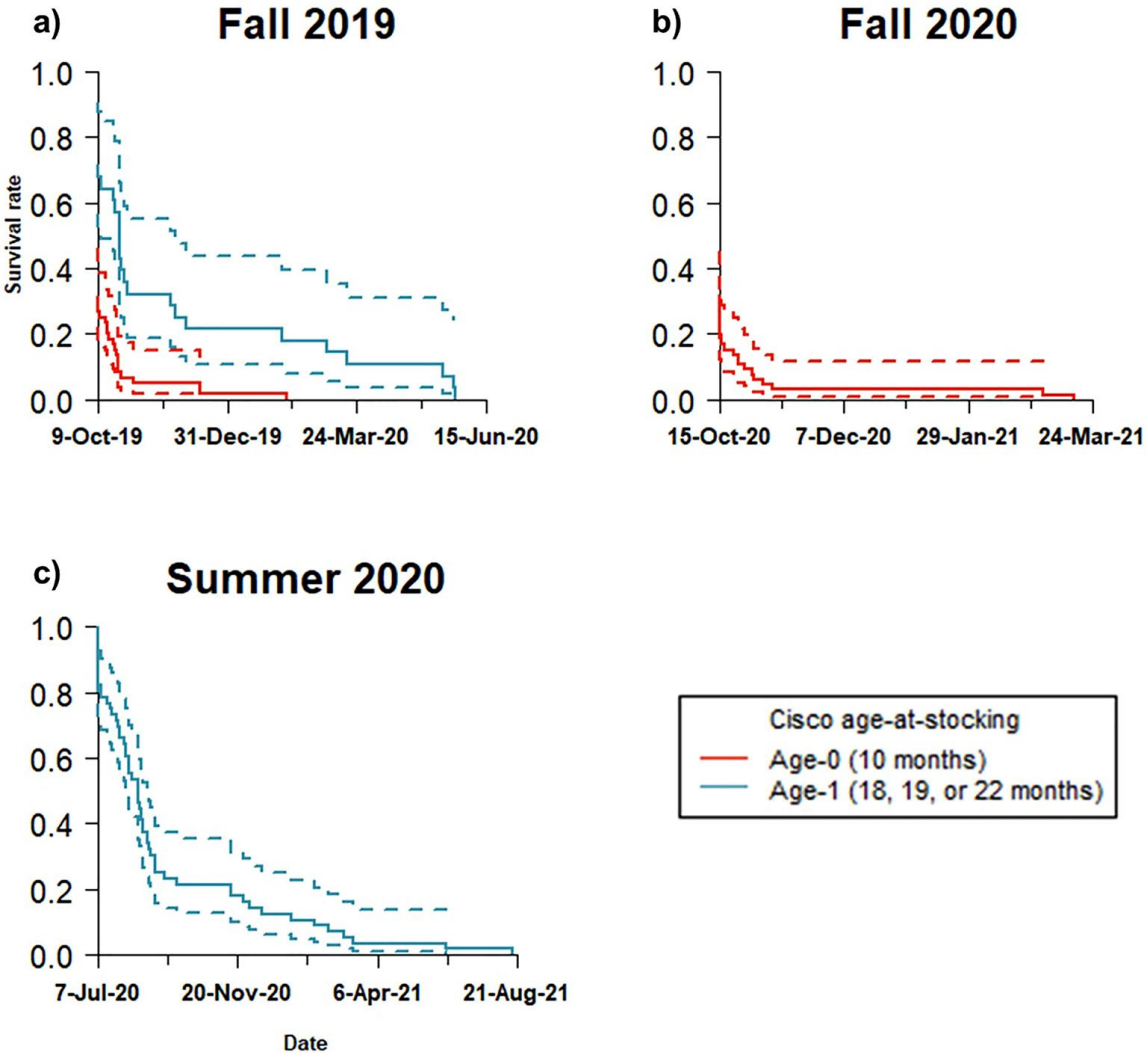
Kaplan–Meier survival curves. Kaplan Meier curves for tagged cisco (*Coregonus artedi*) with estimated survival probability (solid lines) and 95% confidence interval (dashed lines) for each cohort of stocked fish: (**a**) Fall 2019, (**b**) Fall 2020, and (**c**) Summer 2020. Blue lines indicate the age-0 release cohort (Fall 2019, Fall 2020) and orange lines indicate the age-1 release cohort (Fall 2019, Summer 2020). The first dates on the x-axes are the stocking date for each cohort of released fish.

Tagged cisco moved widely throughout Keuka Lake, with detections confirmed at all receivers in the whole-lake acoustic array. Cisco exhibited preference for some regions of Keuka Lake, with the most tagged fish activity found in the northwestern branch (near the stocking site) to the receiver gate midway in the south branch of the lake (Fig. 1). A lower frequency of tag detections was observed in the northeastern branch and at the confluence in the middle of the lake (Fig. 1). The mean time between successive detections for tagged fish at large was 1.92 days (se ± 0.17; range 0–10.4 days). Empirical Kaplan–Meier survival curves indicate high initial mortality at release and sustained high mortality before a few individuals transitioned to a long-term low mortality regime (Fig. 3a–c). Age-1 cisco show substantially higher initial and long-term survival compared to age-0 stocked cisco (Table 2). The maximum observed survival of tagged age-1 fish was estimated as 405 days after release, whereas maximum observed survival of tagged age-0 fish was estimated as 152 days after release.

**Table 2:** Summary of the fate of tagged juvenile cisco released into Keuka Lake, New York, USA from October 2019 to October 2020.

Release cohort (age)	Number of tagged fish released	Number of mortalities within one day (%)	Median time survived (days)	Maximum time survived (days)
Fall 2019 (1)	28	9 (32.1%)	14	230
Fall 2019 (0)	60	45 (75.0%)	0	122
Summer 2020 (1)	56	10 (17.9%)	39	405
Fall 2020 (0)	66	55 (83.3%)	0	152

### Time-to-event survival models

The candidate model set investigated the effects of individual-level covariates on survivorship. We did not detect any structural deficiencies in fitted Cox models, indicating that these regressions satisfied goodness-of-fit testing (see SI 2.1)^23,25^. Visual inspection of Schoenfeld and Martingale residuals of each covariate tested in our most general Cox proportional hazards models only showed minor deviations from β = 0, and deviance residuals failed to indicate highly influential observations. We therefore proceeded with multi-model inference to evaluate the strength of support for each candidate Cox survival models.

**Figure 4:**
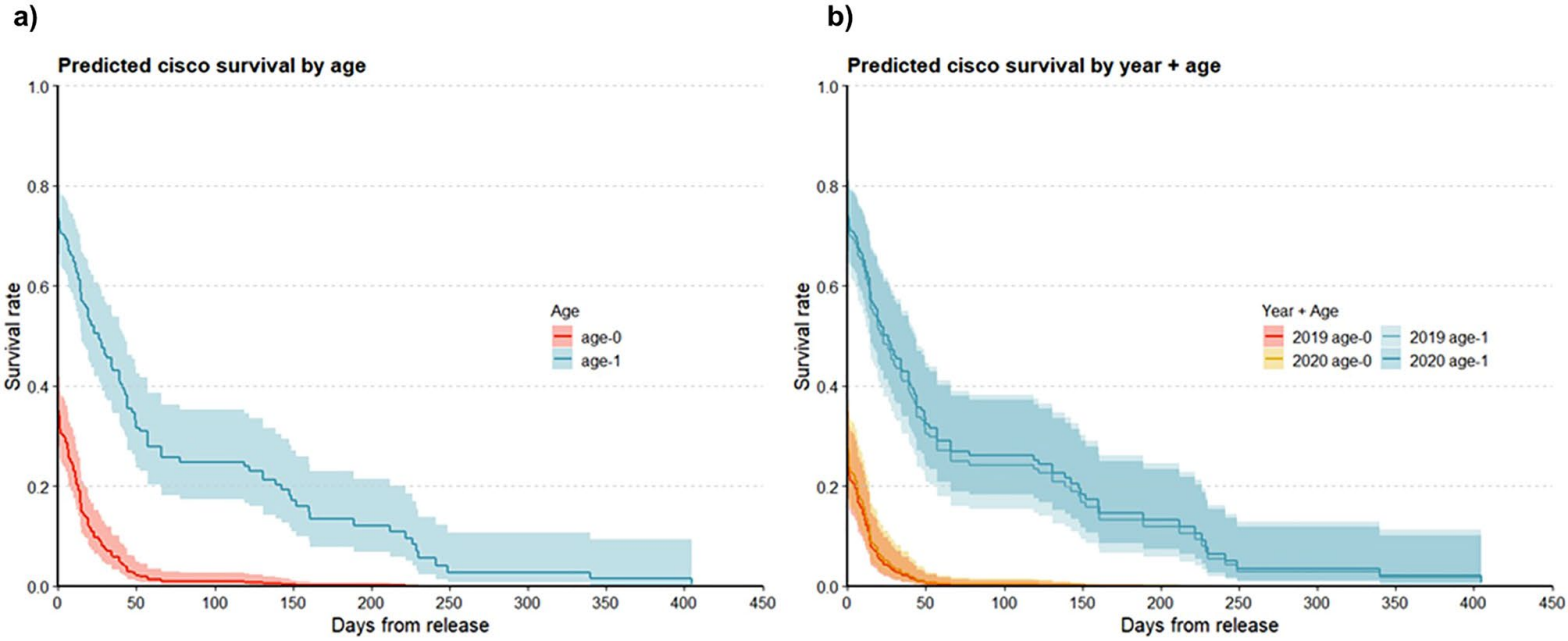
Predicted survival from Cox proportional hazards models. Estimated Cox proportional hazard model survivorship curves for acoustic-tagged juvenile cisco stocked into Keuka Lake, New York, USA. (**a**) Predicted survival and 95% confidence limits (shaded areas) by age-at-release from the top ranked model (Survival ~ Age; 42% model weight) based on Akaike’s Information Criterion as adjusted for small sample size (AICc). (**b**) Predicted survival from the second AICc-ranked Cox model (Survival ~ Year + Age; 17% model weight).

Multi-model inference results highlighted the importance of age-at-release on juvenile cisco survival. The top AICc-ranked Cox model included a single covariate: age-at-release (Table 3; k = 1, AICc 1753.95, and 42% AICc weight). The next top-ranked model within 2 ΔAICc^52^, includes the covariates year and age-at-release (k = 2, 1.87 ΔAICc, 16% AICc weight). Additionally, we found moderate support for a model that included age and condition (k = 2, 2.03 ΔAICc, 15% AICc weight). Our three top AICc-ranked models account for 76% of total model set support by AICc weight (Table 3). Combined across the model set, age-at-release had > 99% relative AICc variable importance, followed by year (28%), condition (28%), length (13%), and mass (< 1%) (Table 4). The top AICc-ranked Cox model, Survival ~ Age, reveals significant differences in predicted survival, $\widehat{S}$, between tagged fish released at age-0 versus at age-1 (Log-rank test for the model *p* < 0.01, χ^2^ = 85.16, 1 *df*; Fig. 4). For example, the estimated coefficient, $\widehat{\beta }$, for age in this model and the corresponding hazard ratio, ${e}^{\widehat{\beta }}$, revealed that tagged age-1 fish experience 75% less mortality than tagged age-0 fish ($\widehat{\beta }$_*age-1*_ =  − 1.39, ${e}^{\widehat{\beta }}$ = 0.25, and 95% confidence limits = 0.18, 0.34). While stocking year and condition factor featured in the next top AICc-ranked models and had moderate relative AICc variable importance, neither variable was found to be statistically significant (*p* > 0.05) in any of the fitted models in which they were included. This indicates that although we observed minor differences in predicted survival given release year and condition factor, age-at-release still emerges as a top predictor of fish survival.

**Table 3:** Candidate model set for Cox proportional hazards regressions fit to time-to-event data from acoustic-tagged juvenile cisco in Keuka Lake, New York, USA.

Cox model structure	k	AICc	ΔAICc	AICc Wt.	Log-likelihood	Cum. Wt.
Age	1	1753.95	0.00	0.42	− 875.96	0.42
Year + age	2	1755.81	1.87	0.16	− 875.88	0.58
Age + Condition	2	1755.98	2.03	0.15	− 875.96	0.73
Length + length:age	2	1756.96	3.01	0.09	− 876.45	0.82
Year + age + condition	3	1757.83	3.88	0.06	− 875.86	0.88
Age + condition + condition*age	3	1758.03	4.09	0.05	− 875.96	0.93
Year + length + length:age	3	1758.62	4.67	0.04	− 876.25	0.97
Year + age + condition + condition*age	4	1759.83	5.89	0.02	− 875.82	1.00
Year + mass + mass:age	3	1764.01	10.06	< 0.01	− 878.95	1.00
Mass + mass:age	2	1766.23	12.29	< 0.01	− 881.09	1.00
Year + length	2	1771.75	17.81	< 0.01	− 883.85	1.00
Length	1	1771.98	18.03	< 0.01	− 884.98	1.00
Year + mass	2	1784.79	30.84	< 0.01	− 890.36	1.00
Mass	1	1788.94	34.99	< 0.01	− 893.46	1.00
Condition	1	1817.95	64.00	< 0.01	− 907.96	1.00
Year + condition	2	1818.03	64.08	< 0.01	− 906.99	1.00
Year	1	1830.01	76.06	< 0.01	− 914.00	1.00

Multimodel inference is based on Akaike’s Information Criterion adjusted for small sample sizes (AICc).

**Table 4:** Relative variable importance values from multi-model inference for Cox proportional hazards models fit to tagged juvenile cisco based on Akaike’s Information Criterion adjusted for small sample sizes (AICc).

Variable	Type	AICc relative variable importance (%)
Age (0, 1)	Categorical	> 99
Year (2019, 2020)	Categorical	28
condition factor (*k*)	Continuous	28
Total length (mm)	Continuous	13
Mass (g)	Categorical	< 1

### Stocked juvenile cisco survival dynamics

Cox survival models indicate long-term survival of few juvenile cisco after initial release into Keuka Lake. Predicted survival from top AICc-ranked Cox models indicate age-1 cohorts have higher immediate post-release survival [time (*t*) < 1-day] and longer overall survival than age-0 cohorts (Fig. 4). For example, survival curves predict high initial mortality within the first day of release (*t* < 1 day), particularly with age-0 cisco which experienced 77% initial mortality (${\widehat{S}}_{(t=1)}$= 0.23), in contrast to 30% initial mortality (${\widehat{S}}_{(t=1)}$= 0.70) with age-1 cisco (Table 2; Fig. 4). From the top-ranked Cox model, Survival ~ Age, the probability of an age-1 juvenile cisco surviving a full year [i.e., ${\widehat{S}}_{(t=365)}$] was 0.02 (95% confidence limits =  < 0.001, 0.099), whereas the annual survival rate predicted for stocked age-0 cisco asymptotes at zero.

## Discussion

Whole-lake biotelemetry coupled with contemporary tag technology enables novel opportunities to estimate survival of juvenile and small fish released into natural aquatic systems. In Keuka Lake, time-to-event models provided survival estimates for stocked cisco and generated insights needed to assess the efficacy of native species reintroduction efforts. By evaluating the time path of survival for stocked cisco, time-to-event data provide insight into fine-scale temporal patterns in survival beyond approaches that assess an overall survival rate over a fixed time interval such as through binomial regression^23,25^. For example, Cox models fitted to stocked cisco data in Keuka Lake reveal that juvenile fish mortality was highest during the initial post-stocking period (within one day of release), and that longer term survival was dependent on age-at-release. Survival models indicated that mortality within the first day after release was particularly significant for age-0 fish (< 25% survival) compared to age-1 stocked fish (70% survival). These results provide fishery managers with an indication of the effect of using different sized hatchery fish on the likely success of restoring a native cisco population.

The high mortality upon release of juvenile cisco observed in Keuka Lake may stem from a suite of potential factors, including avian predation, predation by lake trout (an abundant top predator in the lake), and physiological stress. Avian predation was witnessed by project biologists at stocking, and fish predators were observed on hydroacoustic units from boats during stocking. Local anglers captured two lake trout with two depredated tagged cisco found in stomach contents shortly after stocking, providing anecdotal but direct evidence of high potential for piscivore predation (SI 3.1; Fig. S6). Reduced survival from handling, transport, and stocking of hatchery-reared fish is well-documented^1,13^, and these factors could also have contributed to mortality from physiological stress for stocked cisco in this study. Tag burden could have also contributed to mortality (see SI 1.1); however, we followed protocols predicted to lead to low tagging-related mortality according to a study design to evaluate juvenile cisco response to JSATS tag implantation^33^. Further, we monitored tagged fish at the hatchery prior to release and did not find evidence of poor condition or mortality from tagging, suggesting that tag burden may not have been a significant source of stocking mortality. Moving forward, we anticipate opportunities to use predation sensor tags^9,38^ or to assess survival outcomes for cohorts released under alternative stocking practices that could help disentangle and quantify specific mortality drivers for stocked juvenile fish.

We found that juvenile cisco in both age classes survived the initial release then experienced an acclimation period of elevated mortality, with a midpoint of this acclimation period of approximately *t* = 30 days after release for age-0 fish and approximately *t* = 50 days for age-1 fish. Although no age-0 fish survived greater than six months after release, we found approximately 15% survival rate to six months in age-1 fish, with only one age-1 fish surviving more than one year after stocking (2% annual survival rate). While this is the first study to yield survival estimates for this species with time-to-event data, comparable studies elsewhere in the Great Lakes region of North America estimate low survival rates of other coregonine species such as bloater (*Coregonus hoyi*). For example, population modeling and trawl surveys estimate < 20% initial apparent survival of stocked juvenile bloater^27^ and low initial apparent survival (≤ 42%) of tagged age-1 bloater through acoustic telemetry in Lake Ontario^53^. From our survival analysis, few individuals survived to the end of the study, and therefore it is unlikely that enough cisco from those experimental releases survived to reproductive maturity, which would be required to establish a self-sustaining population in Keuka Lake. Nonetheless, mortality rates for both age classes appeared to decrease and stabilize after approximately *t* = 60 days. This suggests that if fish survive through an acclimation period of high mortality, remaining individuals might enter a natural mortality regime allowing for some long-term survival.

Time-to-event survival estimation provides insight into predictors of tagged fish mortality by constructing models that investigate the association of subject-level covariates with estimated hazard rates for mortality. Our top-ranked Cox proportional hazards models predict higher initial survival and longer-term survival for age-1 stocked fish than for age-0 stocked fish. These results may indicate that there is a size threshold for stocked juvenile cisco needed to escape high rates of predation from birds and fishes^54,55^. Additionally, large fish may have higher energy reserves to persist through physiological stress at release, as well as to survive through a period of famine as stocked fish adapt to lake foraging conditions^56–58^. Such factors could have limited the ability of juvenile cisco, particularly age-0 fish, in this study to acclimate to lake depths, navigate to suitable habitats, or find suitable prey sources for feeding^29,53^. Future experiments could thus include stocking large juvenile cisco as a possible mechanism for predation avoidance via predator gape limitation and to survive through acclimation of the lake environment.

Post-release survival results for stocked juvenile cisco in Keuka Lake highlight the importance of evaluating stocking outcomes through the fate of hatchery-reared fish inferred from acoustic telemetry. We found that a small proportion of stocked age-0 cisco survived through early stages after their release, comparable to similar findings of acoustic-tagged juvenile bloater^53^. Results indicate that age-1 cisco experience higher survival than age-0 stocked fish, which aligns with results from other studies^59,60^. Rearing pelagic schooling fish, such as cisco, to age-1 or greater requires significant hatchery space and is costly at a sufficiently large scale. Instead, survival curves estimated for juvenile stocked fish in Keuka Lake suggest that alternative release practices may improve chances of cisco restoration success. For example, applying estimates from the top AICc-ranked fitted Cox model (Age-0 cisco ${\widehat{S}}_{(t=1)}$= 0.23, and assuming a fixed long-term survival rate of the upper 95% confidence limit ${\widehat{S}}_{(t=180)}$= 0.004), a 25% reduction in first day post-release mortality would lead to a predicted 83% increase in survival of age-0 fish to six months. Reducing initial mortality through alternative stocking practices shows promise in increasing the number of fish that survive through high mortality periods and into a long-term survival regime to potentially reach reproductive maturity^1,13^.

In addition to survival, detection histories can also provide insight into movement and habitat use of tagged fish. Sections of the lake between receivers lacked detection coverage, and thus fine-scale spatial insights were beyond the scope of this study. Generally, we found that tagged cisco surviving past initial release moved widely throughout the lake with preference by both age classes for the west and south branches of Keuka Lake. Tagged age-0 fish that survived *t* ≥ 30 days (n = 4) were detected on 8.3 receivers on average (range 4, 14) and age-1 fish that survived *t* ≥ 50 days were detected on 7.9 receivers on average (range 1, 19), which demonstrates the spatial ecology utility of acoustic receiver arrays. Tagged cisco that survived high post-release mortality to an acclimation period therefore exhibited their expected high movement behavior^31,37^.

Survival estimates from this study can be used to adapt stocking practices and improve the probability of successfully achieving management goals for cisco restoration^28^. Our approach provides continuous survival monitoring from the onset of this reintroduction effort, as opposed to evaluating success after multiple years of stocking via detection or capture of surviving adults. In Keuka Lake, acoustic telemetry revealed that survival rates are too low under current stocking practices to re-establish native forage populations, thus informing updates to stocking practices to reduce stocked fish mortality. Managers sought stocking age-0 cisco after the lake de-stratified in October to allow lake trout to distribute throughout the water column, thereby overwhelming predators with numerous prey targets^27^. Several hatchery release practices may show promise in reducing initial mortality of stocked juvenile fish. Net pen stocking—where fish are held for a period of weeks in situ in enclosed pens and provided feed to assist in transition to novel environmental conditions—may assist juvenile cisco in averting high initial predation, facilitate acclimation to lake conditions, and transition to wild defensive behaviors such as schooling^61^. For example, in both marine and freshwater fisheries, net pen-stocking has been used for stocking several Salmonine species including Pacific salmon (*Oncorhynchus spp.*)^1^. In addition to net pen-stocking, stocking at multiple release sites and at night may improve survival outcomes by assisting stocked fish in avoiding predator gauntlets. Previous studies have shown that cisco have a predator-driven diel migration^26,53^, which could potentially be leveraged to mitigate juvenile cisco overlap with predatory birds and fish through nighttime releases but requires investigation.

This study shows that acoustic telemetry technology can track the fate of tagged fish in a deep inland lake. Our approach may be well-suited for supporting fisheries research and conservation efforts in such inland lake settings which are widely distributed across many landscapes. While this technology may be scalable to waterbodies of similar depth and area, it may be unfeasible to deploy arrays at sufficient density to meet survival modeling assumptions used here in larger lakes or marine settings. In settings that fail to meet closed population assumptions (e.g., tagged fish can leave the area monitored by a receiver array), mortality and apparent survival estimates are often calculated from tracking electronically tagged fish through a series of spatially coordinated acoustic receivers whereby directional swim rates between receivers, constant tag transmissions, or movement off the array are incorporated along with detection probability^24,53,62,63^. Nevertheless, telemetry receiver coverage has continued expanding in larger lake settings such as the Great Lakes, USA and Canada and Lake Champlain, USA as well as marine settings along the Pacific and Atlantic Coasts, USA and Canada (e.g., Atlantic Cooperative Telemetry Network, Pacific Ocean Shelf Tracking project) and Australia (e.g., Integrated Marine Observing System in Australia)^8,64^. As acoustic technology advances, receiver network coverage will continue to provide finer spatial and temporal coverage of life history stages of tagged fish^21^.

Results from the Keuka Lake whole-lake biotelemetry study demonstrate that novel acoustic technology coupled with time-to-event modeling provided valuable estimates of survival rates of tagged juvenile fish. While long-term monitoring requires maintenance of acoustic receiver arrays for extended periods, time-to-event models are well suited to such data, accommodating censorship events that may arise from field challenges. For example, time-to-event models can produce unbiased survival estimates even in the presence of ‘interval’ censor events. Such events can arise if fish perish during windows of time when acoustic receiver arrays may be inoperable or inaccessible due to field conditions or equipment failures. While previous tag size restricted telemetry applications to relatively larger fish, small acoustic tags now have sufficient battery life to monitor juvenile fish outcomes for periods of months or longer, as demonstrated in this study. In addition to tag miniaturization, emerging biotelemetry technology can capture additional information useful for disentangling sources of mortalities, such as depth and temperature^14,15^, acceleration^65,66^, and depredation^38^ data. We anticipate growing application for biotelemetry to estimate fish survival in systems for which whole-waterbody acoustic receiver array coverage with known population closure can be maintained for periods spanning entire life cycles.

### Supplementary Information

Supplementary Information.

## Data Availability

Data and code used for all analyses here, developed using R version 4.2.2, are available upon request from the corresponding author. At the time of publication, data were not publicly available from New York State Department of Environmental Conservation.
