# Supplementary material for: Whole-lake acoustic telemetry to evaluate survival of stocked juvenile fish
Source: Sci Rep. 2023 Nov 2;13:18956. doi: 10.1038/s41598-023-46330-6 (PMC10622427; doi:10.1038/s41598-023-46330-6)
Supplement: Supplementary file 1 — Supplementary Information. [file 41598_2023_46330_MOESM1_ESM.pdf]

Whole-lake acoustic telemetry to evaluate survival of stocked juvenile fish

Alexander L. Koeberle[1], Webster Pearsall[2], Brad E. Hammers[2], Daniel Mulhall[2], James E. McKenna, Jr.[3], Marc Chalupnicki[3], Suresh A. Sethi[1,4]

[1]New York Cooperative Fish and Wildlife Research Unit, Department of Natural Resources and the Environment, Fernow Hall, Cornell University, Ithaca, New York 14853, USA

[2]New York State Department of Environmental Conservation, Division of Fish, Wildlife, and Marine Resources, Region 8 – Rochester/Western Finger Lakes, Avon, New York 14414, USA

[3]U.S. Geological Survey, Great Lakes Science Center, Tunison Laboratory of Aquatic Science, 3075 Gracie Road, Cortland, New York 13045, USA

[4]Aquatic Research and Environmental Assessment Center, Earth and Environmental Sciences, 123 Ingersoll Hall, Brooklyn College, Brooklyn, NY 11210

Correspondence: Alexander L. Koeberle, New York Cooperative Fish and Wildlife Research Unit, Department of Natural Resources and the Environment, Fernow Hall, Cornell University, Ithaca, New York 14853, USA, Email: alk239@cornell.edu

S1. Methods

S1.1. Acoustic-tagging and hatchery practices

S1.1.1. Hatchery rearing

Staff from U.S. Geological Survey (USGS) and New York State Department of Environmental Conservation (NYSDEC) collected cisco (*Coregonus artedi*) brood stock annually from Lake Ontario in Chaumont Bay, New York, USA during the spawning season (late November to early December) and transported 200-300+ adult specimens to USGS Great Lakes Science Center, Tunison Lab of Aquatic Science (USGS-Tunison) in Cortland, New York, USA for hatchery propagation. Adults were spawned over the next one to three days and eggs were transferred to incubation tubes to develop. In total, approximately 300,000-1,000,000 total eggs were collected and reared annually at regional Finger Lakes hatcheries including NYSDEC Bath and Oneida New York State Fish Hatcheries (Bath and Oneida Hatcheries) and USGS-Tunison.

S1.1.2. Tagging surgeries

Surgeries to implant acoustic telemetry transmitters (hereafter, ‘tags’) were conducted by staff at USGS-Tunison and followed protocol by McKenna et al. [1]. Age-0 cisco were surgically implanted with small acoustic tags (0.6g Lotek model L-AMT-5.1B; Lotek, Newmarket, Ontario, Canada). Age-1 cisco were implanted with larger acoustic tags (3.5g Lotek model L-AMT-8.2). Full tag specifications are available here: https://www.lotek.com/wp-content/uploads/2017/10/JSATS-AMT-Series-Spec-Sheet.pdf). For all tagged juvenile cisco released in this study, fish were placed on the surgery table and anesthetized with MS-222 solution over the gills via a peristaltic pump (Cole-Palmer Masterflex L/S; 25 mL/minute rate). Physical tags were inserted through a small incision in the abdomen, anterior to the left pelvic fin. Wounds were closed with a single surgeon’s knot using blue monofilament 4-0 suture

(Matrix Wizard LLC). Mean surgery time for 0.6g tags in the McKenna et al. (2021) study was 100.3s (Standard Error, SE, 2.56). For tagged fish released into Keuka Lake, Relative Tag Weight (RTW) for age-0 fish (0.6g tag) was mean = 5.6% (range 3.8-10.9%) and for age-1 fish (3.5g tag) was mean = 5.8% (range 2.2-10.1%). Further details on surgery procedures are available in McKenna et al. [1].

S1.1.3. Transport and stocking

All juvenile cisco (tagged and untagged) were transported to Keuka Lake, New York, USA for release in specialized fish transport trucks with six insulated transport tanks (275-gallon capacity) in each truck equipped with aeration and oxygen diffusion. Each insulated tank could hold up to approximately 7,000 fish per tank and total transport time was approximately 1.5 hours from hatchery to the release site. In total, 296, 979 juvenile cisco were stocked from October 2019 to October 2020 (including 210 tagged fish), with the highest number of fish stocked in fall, e.g., 92,225 age-0 fish stocked on 9-Oct-2019 and 204,466 age-0 fish on 15-Oct-2020). Once on site, fish were directly transferred via 20cm stocking hose from the stocking truck into insulated stocking tanks (also 275-gallon capacity) positioned on a pontoon boat. One tank from the stocking truck was divided between the two stocking tanks on the pontoon boat. Aeration was provided during the five-minute boat trip to the stocking site. Fish were released via a 20cm standpipe that was removed from the bottom of the stocking tank allowing the fish to be discharged directly though the bottom of the boat into the water. No hose was needed for this operation. All fish (tagged and untagged) were stocked above the receiver location coordinates (within 50m at the surface) in the northwestern corner of Keuka Lake. Fish were released manually from the boat using this process to help reduce transport stress as fish are not manually netted out of the tanks and fish can be released volitionally.

S1.2. Acoustic receiver array

Acoustic telemetry receivers (Lotek WHS 4250 Series) were deployed at a whole-lake scale throughout the duration of this study (full receiver specifications available here: https://www.lotek.com/wp-content/uploads/2017/10/WHS-4250-Spec-Sheet.pdf). Receivers were deployed at a lake-wide scale, spaced apart at a mean distance of 2,968.4m (range 1,353m – 6,087m). Range testing was conducted to place receivers that would maximize their detection range, specifically by utilizing three receivers staged at pinch points within each of the lake arms. Receivers at these points were spaced at a mean distance of 240.5m apart (range 184m – 386m). The average sampling depth (range) for acoustic receivers by branch was West = 50m (44-57m), Confluence = 35m, South = 43m (32-52m), and East = 30m (16-37m).

Acoustic receiver range testing (receiver efficiency) was conducted on several days in September and October, prior to the release of tagged fish. The stated manufacturer range for receivers used in this study was 50-250m. We evaluated efficiency for conditions in this study by placing three receivers on the lake bottom with 43 Juvenile Salmon Acoustic Telemetry System (JSATS) transmitters in mesh bags deployed at equal 25m intervals over distances 25-800m from receiver locations. An additional test was conducted with one receiver and two tags deployed at incremental distances out to 300m. Tags were programmed on a 20-second transmission rate (the same rate as used for tagged fish in this study) tied to a moored buoy at the surface at fixed distances and left to transmit for 30 minutes. All gear was picked up and the data were analyzed, whereby we found a receiver efficiency of approximately 200-300m (linear diameter). We found all tags were detected within this range, with an immediate lack of detections for tags positioned further than these distances (i.e., a distance threshold for tag detections).

We expected that receiver efficiency would increase at greater depths (closer to the receiver), with surface detections being the farthest extent of each receiver's coverage. Further, the water column mixes from late fall through early summer (e.g., no stratification), so we do not anticipate detection efficiency would be limited by depth during this period [2]. Despite stratification with differences in water temperature and density (e.g., the summer thermocline) that may cause detection deflection or interference, we expected cisco to primarily inhabit the cooler waters below the thermocline during this period.

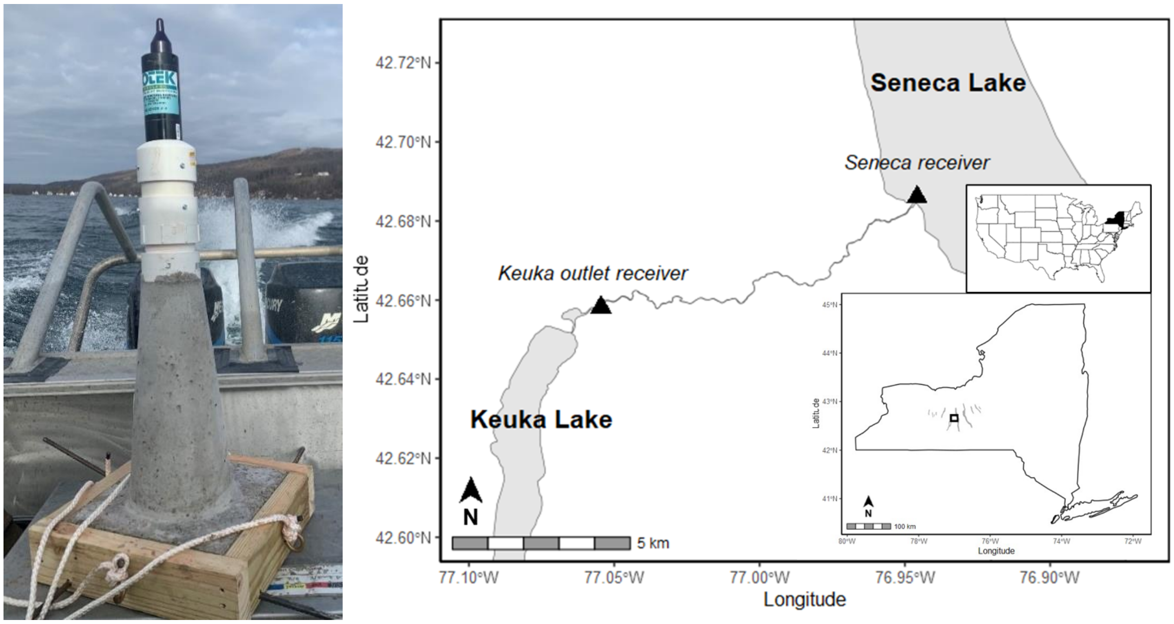

Figure S1. (**Left**) Example of an acoustic receiver mooring (using Lotek-brand Wireless Hydrophone System (WHS) 4250 Series Juvenile Salmon Acoustic Telemetry System (JSATS) Acoustic Autonomous Receivers/Dataloggers. The acoustic receiver is anchored approximately one meter from the bottom of the cement block and is facing upward to detect acoustic tagged cisco (*Coregonus artedi*) swimming in the water column. Each mooring also contained a cable anchor for retrieval and surface buoy for safety and identification. All acoustic receivers across the array were deployed with this configuration across Keuka Lake, New York, USA. Photo by D. Mulhall. (**Right**) Location of acoustic receivers placed in the Keuka Lake outlet (receiver depth = 1.8m) and nearby Seneca Lake (receiver depth = 1.8m) to detect potential emigration of tagged juvenile cisco. The outlet flows from the northeastern branch of Keuka Lake eastward into Seneca Lake. No tagged cisco were detected on either receiver for the duration of this study. Prior to this study, we did not expect cisco to utilize this habitat as the Keuka Lake outlet is

shallow and contains a natural waterfall barrier midway that likely prohibits fish emigration to Seneca Lake.

S1.3. Detection filtering and assumptions

S1.3.1. Detection criteria

All tag detection filtering was conducted using statistical software (program R; R Core Team, 2022) with output datafiles from Lotek WHS Host software (V1.8.3942.3) that were filtered to only include entries that corresponded to unique fish tag identifications. In the software program (program R), we took the following steps to validate true tag detections:

1) Calculate the time (*t*) between consecutive tag detections for each fish by applying the ‘min_lag’ function from the ‘GLATOS’ R package [3].
2) False detections were then omitted from the acoustic telemetry dataset based on the following criteria:
    a. All detections must occur after time of stocking and before the minimum (80%) expected battery lifespan stated by the manufacturer (Lotek-brand): 0.6g tag = 262 days and 3.5g tag = 1,218 days.
        i. Maximum tag detections were observed on the acoustic receiver array for age-0 fish (0.6g tags) as 151 days (n = 1 tagged fish; 57.6% of minimum expected battery life), and for age-1 fish (3.5g tags) as 403 days (n = 1 tagged fish; 33.1% of minimum expected battery life).
    b. A specific function (the ‘false_detections’ function from GLATOS) was used to specify consecutive detections from ‘min_lag’ must occur within 10 minutes, or

30 times the nominal rate of programmed transmitter [3, 4], e.g., 30*20-second nominal delay = 600s.

c. Final true detections must include ≥ two consecutive tag detections on the same receiver within a four-minute window.

We found that most filtered detections that met this criterion occurred at 20s or 40s intervals (within ± 2s). Observed tag detections that occurred < 18s and between 22s and 38s were considered false detections and were therefore removed. All remaining detections ≤ 10 minutes were retained using the 'false_detections' function. We compared all final detections from our detection filtering criteria in R to manual inspection. Manual inspection of individual tag detection histories included visual examination of abacus plots using the 'abacus_plot' in GLATOS compared with the individual filtered detection files. Abacus plots show individual tagged fish movement at receiver sites over time, thus providing visual insight to biological feasibility of detections across the receiver array [3].

Once we obtained our final detection dataset, we then applied the following criteria to infer initial fish mortality:

1) Undetected: Tagged fish that went undetected (e.g., no positive detections at any receiver after stocking) were inferred as straight-to-death, or mortality upon release, events (t = 0 days).
2) Detected < 1d: All fish with positive detections exclusively within the first day (e.g., $0 < t < 1$ day post-release) had detections that only occurred < 2 hours from their first detection post-stocking. Because fish were stocked in stages, precise release times for individual fish were not recorded at stocking. Therefore, same day detections were

calculated as a fraction of a day from first to final raw detection in the number of minutes (fraction of $t = 1$ day) in our final detection dataset.

The final tag detection (age-1 fish released in July 2020) occurred on our acoustic receiver array on 14 August 2021, with an average time between detections as 3.3 days. Our acoustic receiver array was deployed through 2021 with no tagged cisco detections occurring between the final tag detection and December 2021. Hence, we assumed that no tagged fish were alive in Keuka Lake in our study after 14 August 2021.

S1.3.2. 'Detection events' and 'detection interval'

Given tagged fish could still be at large after their final observed detection on the receiver array, we inferred final survival times by adding a detection interval to raw final detection times. Average time between detection events was calculated using a software package (from the 'detection_events' function in GLATOS). To reduce single detections in our final detection datafiles into detection events, we used the criteria: ≥ four consecutive tag hits within one hour. We then conducted a sensitivity analysis to compare no detection interval (zero interval; raw time), half the average time between detections (half interval), and the full average time between detections (one interval). We selected a half interval and added this calculated time to the final raw true detections, rounded to the nearest whole day.

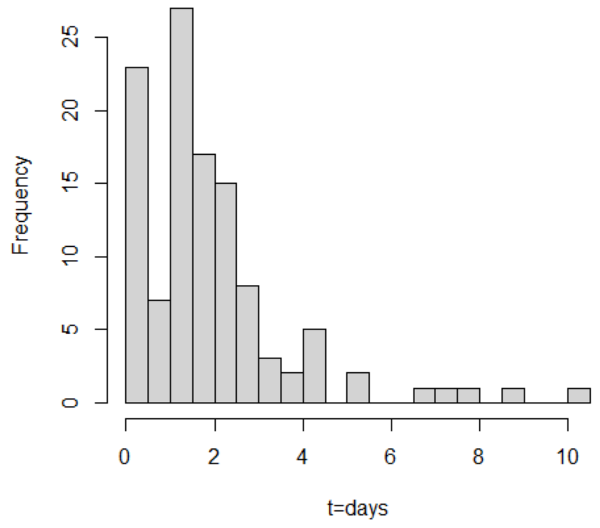

Figure S2. Histogram for calculated average time (days) between detection events for all release cohorts of tagged cisco (*Coregonus artedi*) into Keuka Lake, New York, USA (Fall 2019, Summer 2020, and Fall 2020). Detection interval summary statistics: min=0 days, median=1.52 days, mean=1.92 days, max=10.4 days.

S2. Results

S2.1. Supporting figures for survival analysis

We examined residual plots for the three most general models in our Cox proportional hazards model set: (1) Survival ~ Year + Length + Length:Age, (2) Survival ~ Year + Mass + Mass:Age, and (3) Survival ~ Year + Age + Condition + Condition*Age. We tested hazard assumptions for proportionality based on Hosmer et al. [5] and Moore [6].

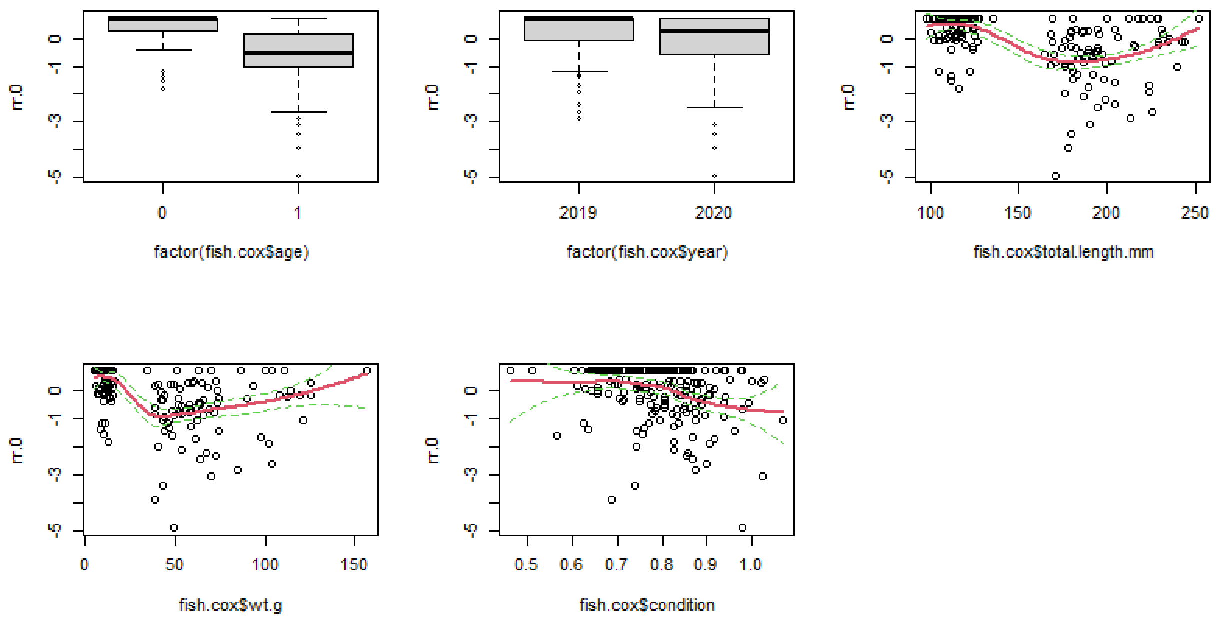

Figure S3. Martingale residuals of a null Cox proportional hazards model plotted against each covariate: Year, Age-at-release, Length, Mass, and Condition factor [6] for tagged cisco (*Coregonus artedi*) stocked into Keuka Lake, NY, USA from 2018 to 2020.

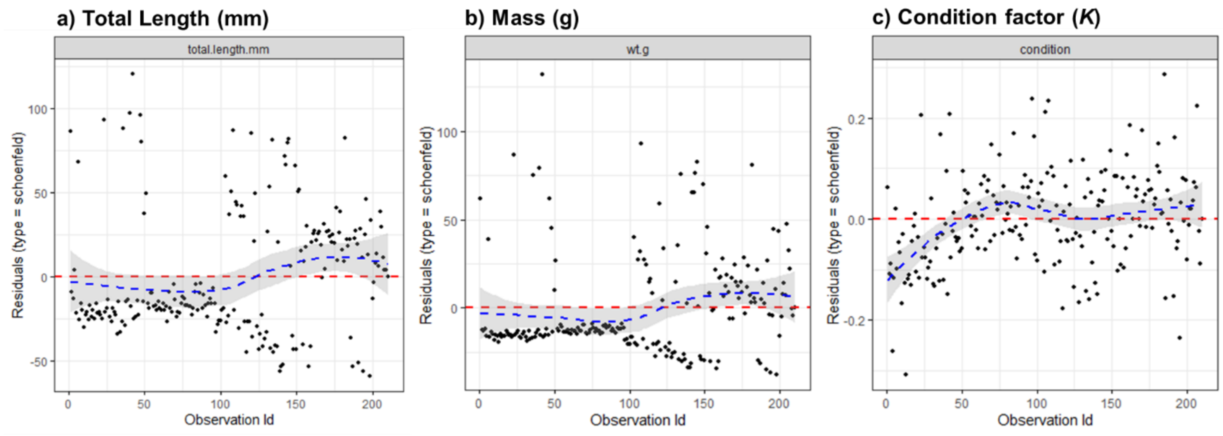

Figure S4. Schoenfeld residuals for subject-level size and condition covariates, **a**) Length, **b**) Mass, and **c**) Condition for tagged cisco (*Coregonus artedi*) stocked into Keuka Lake, NY, USA from 2018 to 2020.

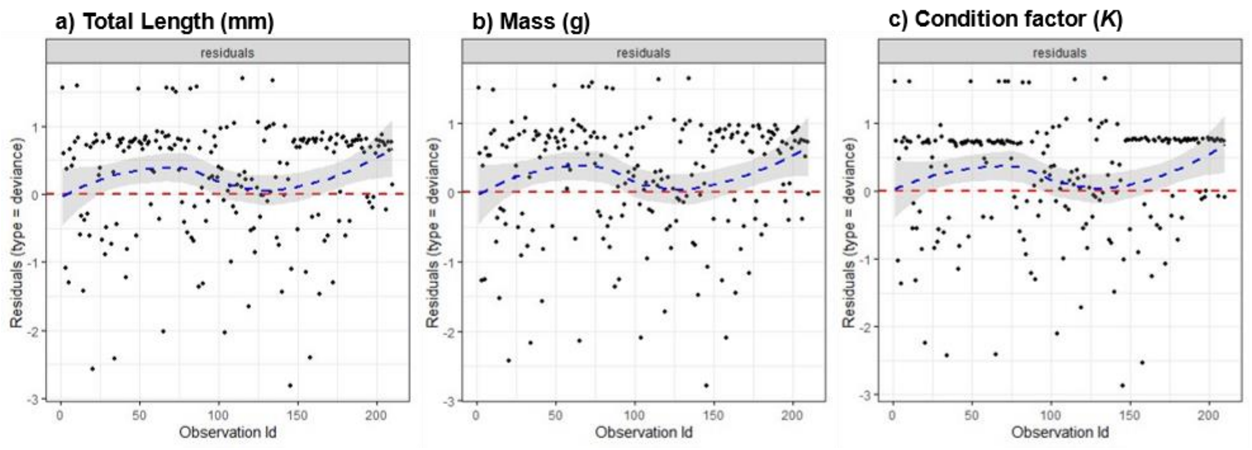

Figure S5. Deviance residuals for each of the most general Cox proportional hazard models: **a**) Survival ~ Year + Length + Length:Age, **b**) Survival ~ Year + Mass + Mass:Age, and **c**) Survival ~ Year + Age + Condition + Condition*Age for tagged cisco (*Coregonus artedi*) stocked into Keuka Lake, NY, USA from 2018 to 2020.

S3. Discussion

S3.1. Evidence of predation

We observed two instances of tagged cisco that were preyed upon by lake trout (*Salvelinus namaycush*), the top piscivorous predators in Keuka Lake. Local anglers caught lake trout with depredated tagged and untagged cisco, with an acoustic transmitter in a lake trout caught in October 2019 and a transmitter in a trout caught in July 2020. The first lake trout was caught two days after fall stocking, with an acoustic transmitter corresponding to an age-0 cisco stocked on 9 October 2019, and detected on the acoustic receiver closest to the stocking site on 10 October 2019. An age-1 cisco was partially digested in a lake trout caught within a week from the 7 July 2020 stocking, with no confirmed transmitter detections on any acoustic receiver in the lake. Both fish had multiple fish in their stomach contents, with a proportion positively identified by project biologists as juvenile cisco. Additionally, a lake trout caught by a local angler on 17 October 2020 (two days post-stocking) contained ≥ 20 juvenile cisco in the stomach contents (no acoustic tags were confirmed inside this trout).

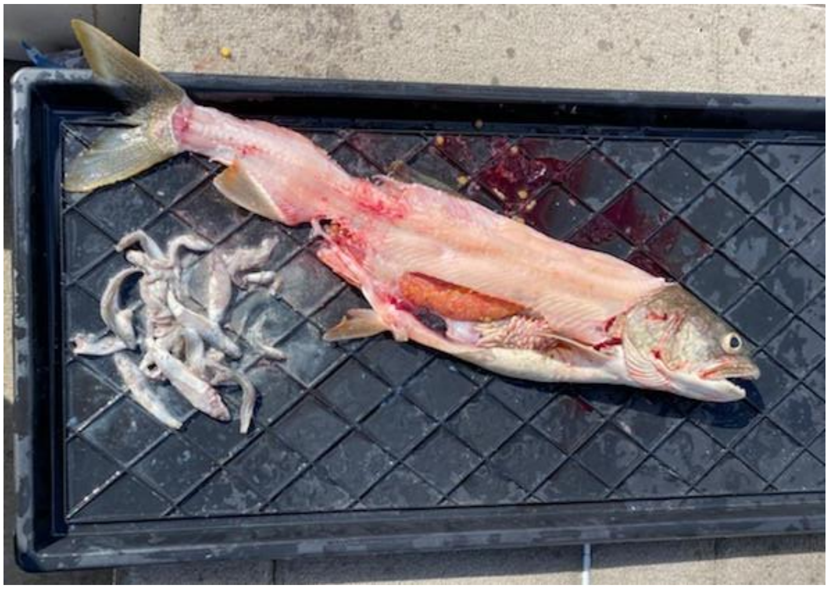

Figure S6. Angler caught lake trout (*Salvelinus namaycush*) on 17 October 2020, two days after fall stocking, with several cisco (*Coregonus artedi*) inside the stomach sample (photo provided to M. Chalupnicki from an anonymous angler).

References

1. McKenna Jr, J. E., Sethi, S. A., Scholten, G. M., Kraus, J. & M. Chalupnicki. Acoustic tag retention and tagging mortality of juvenile cisco *Coregonus artedi*. *J. Great Lakes Res*. **47**, 937-942 (2021).
2. Kuai, Y. *et al*. Strong thermal stratification reduces detection efficiency and range of acoustic telemetry in a large freshwater lake. *Anim. Biotelemetry*. **9**, 46 (2021).
3. Holbrook, C. et al. glatos: A Package for the Great Lakes Acoustic Telemetry Observation System. (2019). <https://gitlab.oceantrack.org/GreatLakes/glatos>.
4. Pincock, D. G. and S.V. Johnston. Acoustic telemetry overview. *Telemetry techniques: a user guide for fisheries research*, 305-337. (2012).
5. Hosmer, D. W., Lemeshow, S. & S. May. *Applied Survival Analysis: Regression Modeling of Time-to-event Data*. Wiley-Interscience 2nd ed. (2008).
6. Moore, D.F. *Applied survival analysis using R*. Springer International Publishing. (2016).
